# Plant Extracts in the Bone Repair Process: A Systematic Review

**DOI:** 10.1155/2019/1296153

**Published:** 2019-11-25

**Authors:** Lyvia Lopes Miranda, Vanessa de Paula Guimarães-Lopes, Luciana Schulthais Altoé, Mariáurea Matias Sarandy, Fabiana Cristina Silveira Alves Melo, Rômulo Dias Novaes, Reggiani Vilela Gonçalves

**Affiliations:** ^1^Department of General Biology, Federal University of Viçosa, Viçosa, Minas Gerais 36570-900, Brazil; ^2^Department of Animal Biology, Federal University of Viçosa, Viçosa, Minas Gerais 36570-900, Brazil; ^3^Institute of Biomedical Sciences, Department of Structural Biology, Federal University of Alfenas, Alfenas, Minas Gerais 37130-001, Brazil

## Abstract

Bone lesions are an important public health problem, with high socioeconomic costs. Bone tissue repair is coordinated by an inflammatory dynamic process mediated by osteoprogenitor cells of the periosteum and endosteum, responsible for the formation of a new bone matrix. Studies using antioxidant products from plants for bone lesion treatment have been growing worldwide. We developed a systematic review to compile the results of works with animal models investigating the anti-inflammatory activity of plant extracts in the treatment of bone lesions and analyze the methodological quality of the studies on this subject. Studies were selected in the PubMed/MEDLINE, Scopus, and Web of Science databases according to the PRISMA statement. The research filters were constructed using three parameters: animal model, bone repair, and plant extracts. 31 full-text articles were recovered from 10 countries. Phytochemical prospecting was reported in 15 studies (48.39%). The most common secondary metabolites were flavonoids, cited in 32.26% studies (*n* = 10). Essential criteria to *in vivo* animal studies were frequently underreported, suggesting publication bias. The animals treated with plant extracts presented positive results in the osteoblastic proliferation, and consequently, this treatment accelerated osteogenic differentiation and bone callus formation, as well as bone fracture repair. Possibly, these results are associated with antioxidant, regenerative, and anti-inflammatory power of the extracts. The absence or incomplete characterization of the animal models, treatment protocols, and phytochemical and toxicity analyses impairs the internal validity of the evidence, making it difficult to determine the effectiveness and safety of plant-derived products in bone repair.

## 1. Introduction

Bone lesions are an important health problem, causing social and financial burden [1]. It is estimated that, with the increase in the elderly population in the world, the incidence of fractures increases even more in the next years [2]. In 2015, costs for fracture treatments were about $17.8 billion, and by 2025, annual costs are expected to exceed $25 billion each year in the United States [3]. Bone remodeling is composed of a complex sequence of cellular events [4] that include phases of inflammation, cell proliferation, and bone remodeling, which is controlled by osteogenesis and angiogenesis [5]. In most cases, bone fractures are caused by specific bone traumas or diseases [6].

The bone has high capacity of remodeling, being able to regenerate and maintain its structure and function. However, there are clinical situations in which the acceleration of bone formation is desirable [7]. Research has been carried out to better understand the inflammatory mechanisms that regulate this repair process and identify new therapeutic targets for the treatment of bone fractures [8, 9]. In this context, natural products, biomaterials, and their derivatives have stood out as a promising alternative to minimize side effects, reduce costs, and promote a fast and efficient repair process [10].

Drugs derived from medicinal plants are consumed by about 75% of the world's population [11] and represent the main form of treatment for traditional medicine in the majority of developing nations [12]. Studies have shown that some molecules extracted from natural compounds have high anti-inflammatory, antioxidant, and regenerative effects, which justifies the successful use of these products in different diseases [13]. However, the mechanisms by which compounds of natural origin act in the inflammatory process and consequently in bone healing are still poorly understood. Current evidences are sparse, fragmented, and based on punctual researches, which makes the results described in the literature inconsistent. Although not clear, we believe that the action of the plant extracts is related to the increase in the antioxidant defenses and decreased tissue inflammation, as well as the increase in the vascularization of the tissue and proliferative activity of the bone cells.

Clinical and preclinical studies have attempted to demonstrate the positive effects of plant compounds on bone matrix formation and cellular activity [14, 15]. However, this proposition is not always confirmed, mainly due to the great methodological variations involving the extract preparation, therapeutic schemes, and mechanisms of action [16]. Therefore, it is necessary to compile data from several studies in order to clarify the previous discrepancies. In this context, we systematically analyzed the preclinical evidence *in vivo*, to establish the relevance of the use of vegetal products in bone repair. In addition, we aimed to determine if there is a rational selection criterion of the plant species to be used and the geographic distribution of each species, as well as any evidence of bioprospection based on ethnobotanical data. We also performed a critical analysis of the studies, aiming to improve the quality of the reports, preventing the reproduction of methodological failures in new studies.

## 2. Materials and Methods

### 2.1. Search Strategy

The PRISMA (Preferred Reporting Items for Systematic Reviews and Meta-Analyses) strategy was applied to identify all studies included in this review [17]. A direct search was carried out from three comprehensive electronic databases: PubMed/MEDLINE, Scopus, and Web of Science. The secondary search was based on the screening of the reference list of all relevant studies identified in the direct search.

Structured search filters were developed for each database. The search filters were initially constructed considering standardized descriptors extracted from PubMed thesaurus (MeSH (Medical Subject Headings)). All descriptors were combined in a complete three-level search strategy based on (i) animal model, (ii) bone repair, and (iii) plant extracts. Standardized descriptors were defined by the MeSH algorithm, and non-MeSH descriptors were characterized by the TIAB algorithm which was also used to recover recently published studies and studies in process for indexation. A previously published and optimized animal filter was applied in a PubMed search interface [18]. The same search filters used for bone repair and intervention were adapted for Scopus. The Scopus' own animal filter (keyword—animals [limit to]) was used in this database. Only studies in English, Portuguese, and Spanish were recovered, and no chronological limits were applied in our search strategy ([Supplementary-material supplementary-material-1]). All relevant studies published until September 10, 2019 (updated search date), were recovered and included in the systematic review.

### 2.2. Record Screening and Eligibility

All research records recovered in the database search were analyzed, and duplicates were removed considering the authors, title, journal, and year of publication. After title and abstract screening, all potentially relevant studies were evaluated in full text for eligibility according to specific inclusion and exclusion criteria. Only original studies investigating the relevance of plant extracts on bone repair in preclinical studies with animal models were included. The exclusion criteria were based on the following: (i) it is not bone, (ii) it is not a plant extract, (iii) laminectomy, (iv) absence of bone defect, (v) peptides and fractions obtained from plants, (vi) compounds obtained from animals, (vii) *in vitro*, (viii) secondary studies (literature reviews, letters to the editor, case studies, comments, and editorials), (ix) marketed products, (x) associated treatment (treatment with plant extracts associated with the other plants and other compounds such as collagen matrix, laser, physical activity, and commercial drugs), (xi) other language, and (xii) bone marrow. Eligibility was independently analyzed by the researchers, and disagreements were resolved by consensus. In order to enhance the comprehension of the research strategy, the reference lists of all relevant papers identified from the database search were screened for additional studies.

### 2.3. Data Extraction

An initial selection based on the title and abstract (TIAB) was conducted by three independent reviewers. In case of disagreements, a fourth reviewer (RVG) decided whether the study met the inclusion and exclusion criteria. In order to discard subjectivity in the data collection and selection strategy, the information was independently extracted by the four reviewers (LLM, LSA, MMS, and RVG) and analyzed separately.

Data were extracted and tabulated in a descriptive way (tables of descriptors and results). The data extraction was categorized as follows: (I) characteristics of publication: author, year, and country; (II) characteristics of the animal model: species, sex, age, and weight; (III) treatment characteristics: total number of animals, number of animals in each group, control group, treatment time, osteoporosis induction, bone type, bone defect model, lesion size, anesthetics, and euthanasia procedure; and (IV) plants: species, used part, popular indication, extraction and purification method, dose, administration, secondary metabolites, and geographical distribution.

### 2.4. Methodological Bias

Reporting bias was analyzed based on methodological requirements described in the ARRIVE (Animal Research: Reporting of *In Vivo* Experiments) guideline [19]. This strategy requires the complete screening of all manuscript sessions (abstract to acknowledgements and funding) to evaluate the completeness of the scientific reports in animal studies. The screening strategy was based on short descriptions of essential characteristics such as baseline measurements, sample size, animal allocation, randomization, experimental concealment, statistical methods, ethnical statement, and generalizability power. A table summarizing all relevant and applicable aspects was designed considering the specificity and aims of the systematic review.

## 3. Results

### 3.1. PRISMA Guideline

**Figure 1:**
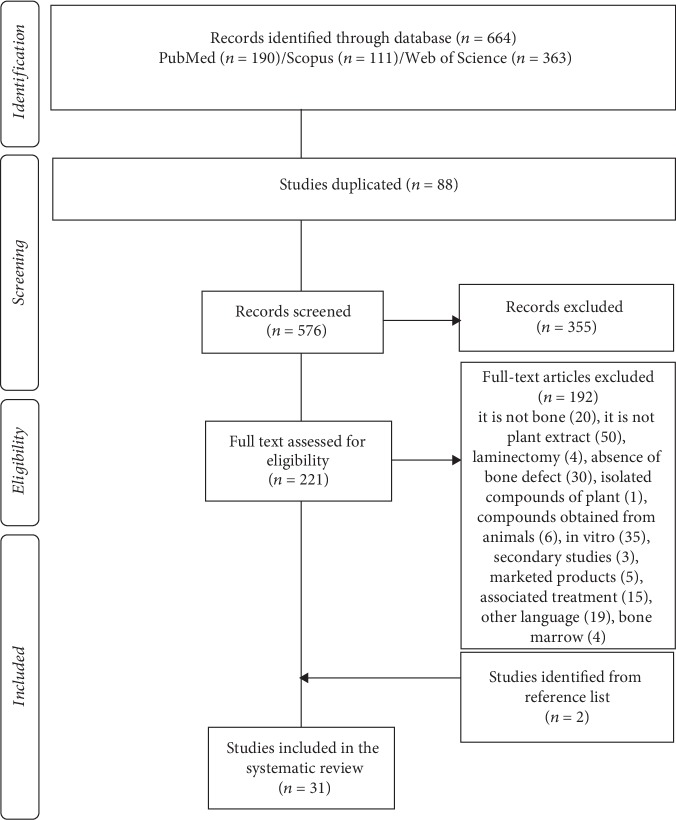
PRISMA diagram. Different phases of selection of studies for conducting qualitative and quantitative analyses. Flow diagram of the systematic review literature search results. Based on “Preferred Reporting Items for Systematic Reviews and Meta-Analyses: The PRISMA Statement” (http://www.prisma-statement.org) [17].

From the PubMed/MEDLINE, Scopus, and Web of Science databases, 664 articles were recovered. A total of 88 duplicated studies and 528 with inadequate thematic were excluded after reading the title and abstract. Of the 48 remaining studies, 19 articles were excluded after reading the full text for not meeting the eligibility criteria. Therefore, 29 studies were included in the systematic review. The reference list of all included studies was analyzed to ensure the identification of additional relevant studies, and 2 of them were recovered, totalizing 31 studies.  Figure 1 shows the flowchart and each step performed in the selection process to recover relevant studies.

### 3.2. Qualitative Analysis

The general characteristics of all included studies are shown in Table 1. The analyzed studies were conducted in 10 different countries especially India (25.81%, *n* = 8), followed by China and Brazil (16.13%, *n* = 5 each), Cameroon and Turkey (9.68%, *n* = 3 each), and Germany (6.45%, *n* = 2). The most commonly used animal models were murine (80.64%, *n* = 25) and rabbits (19.35%, *n* = 6). Considering the animal strain, 51.61% (*n* = 16) were Sprague-Dawley rats; 22.58% (*n* = 7), Wistar rats; and 19.35% (*n* = 6), New Zealand white rabbits, followed by rats and mice (6.45%, *n* = 2 each). From the experimental models, 51.61% (*n* = 16) used female animals, 32.26% (*n* = 10) used males, 3.22% (*n* = 1) used both sexes, and 12.90% (*n* = 4) of all studies did not report this information. The animals' age ranged from 7 weeks to 6 months for rats and 6 months for rabbits, and 32.26% (*n* = 10) of the studies did not report this information. The weight of rats ranged from 150 to 350 g, that of rabbits 1.62 to 5 kg, and that of mice 28 to 33 g, and 6.45% (*n* = 2) of the studies did not report this data.

The most used treatments in the control groups were saline solution (19.35%, *n* = 6), followed by *acacia gum* in aqueous medium (16.13%, *n* = 5), and 9.68% (*n* = 3) reported no treatment. Regarding the treatment time, there was great variation from 10 days (6.45%, *n* = 2) to 24 weeks (3.22%, *n* = 1). Eight studies (25.81%) reported that they had induced osteoporosis in the animals and the method used was ovariectomy (16.13%, *n* = 5) or Glucocorticoid-Induced Osteoporosis Program (GIOP) (3.22%, *n* = 1). The most evaluated bone was the femur (54.84%, *n* = 17) followed by the tibia (25.81%, *n* = 8). The methods used to perform the induction of bone defects were described in 90.32% (*n* = 28) of the studies, and 45.16% (*n* = 14) were performed by insertion of a drill bit. A lesion of 0.8 mm in diameter was created in 29.03% (*n* = 9) of the studies, and 38.70% (*n* = 12) of the studies did not report this information. Regarding the anesthetic procedure, 51.61% (*n* = 16) of the studies used ketamine and xylazine, 9.68% (*n* = 3) used chloral hydrate, and 29.03% (*n* = 9) did not use anesthesia. Most of the studies (70.97%, *n* = 22) did not use medicinal drugs postoperatively. More than half of the studies (51.61%, *n* = 16) did not report the euthanasia procedure of the animals, and 9.68% (*n* = 3) used decapitation under anesthesia. The data cited above can be analyzed in Table 2.

### 3.3. Treatment Characteristics

From the 31 studies, 83.87% (*n* = 26) reported the scientific name of the plant and 16.13% (*n* = 5) cited only the popular name. The most used plant structures were the leaves (25.81%, *n* = 8), followed by the whole plant and roots (9.68%, *n* = 3 each), and 19.35% (*n* = 6) did not record this information. Many authors (19.35%, *n* = 6) did not report the solvent used to extract the components of the plant. Among the studies that presented such information, the most used solvents were ethanol (38.70%, *n* = 12) and a water/ethanol mixture (12.90%, *n* = 4). Most of the studies reported oral administration (*ad libitum*) (51.61%, *n* = 16); however, in 6.45% of the cases (*n* = 2), this information was not reported.

India was the most cited country (9.68%, *n* = 3), in relation to the geographical distribution of plant species, but many studies (61.29%, *n* = 19) did not record this information. In relation to the investigated plants, 48.39% (*n* = 15) realized the phytochemical prospecting, 22.58% (*n* = 7) of them quoted that the phytochemical components were already reported in the literature, and 29.03% (*n* = 9) did not describe this information. The most common secondary metabolites were flavonoids, cited in 32.26% (*n* = 10) of the studies. The anti-inflammatory activity of the extracts was reported in 51.61% (*n* = 16) of the studies, indicating the wide use of plants for the treatments of various diseases (Table 3). The mechanisms of action promoted by plant extracts in the bone repair were neglected in all studies (100%).

### 3.4. Main Parameters Analyzed to Evaluate the Extract Action in Bone Repair

**Figure 2:**
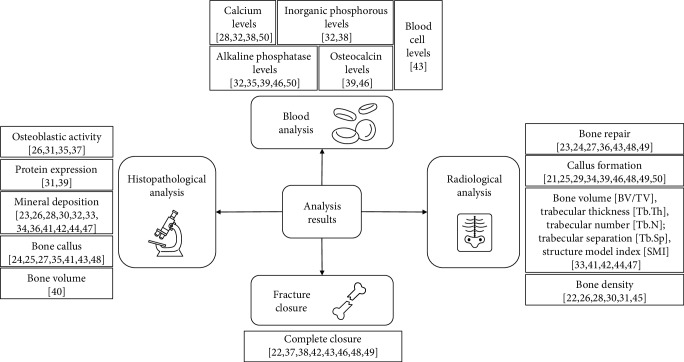
Main results of the studies demonstrating the action of plant extracts in the bone repair process.

The most analyzed parameters among the 31 papers found in this review were radiological analyses (80.64%, *n* = 25) [21–31, 33, 34, 36, 39, 41–50], followed by immunological and histopathological markers (70.97%, *n* = 22) [23–28, 30–37, 39–44, 47, 48]. These analyses demonstrated mainly osteoblastic proliferation, angiogenesis, and increased formation of the bone matrix with fracture closure and bone callus formation. Only 25.81% (*n* = 8) [28, 32, 35, 38, 39, 43, 46, 50] performed measurement of inflammatory markers, and the most cited parameters were Ca^+2^ content and serum alkaline phosphatase. Only 25.81% (*n* = 8) [22, 32, 37, 38, 42, 46, 48, 49] reported whether the fracture had complete, partial, or absent closure. Other analyses related to bone strength, tensile strength, and expression of inflammatory genes that stimulate bone formation and osteogenic differentiation were performed in 38.71% (*n* = 12) of the studies [20, 23, 24, 28, 33, 36, 40–42, 44, 45, 47] (Figure 2).

### 3.5. Bias Analysis

**Figure 3:**
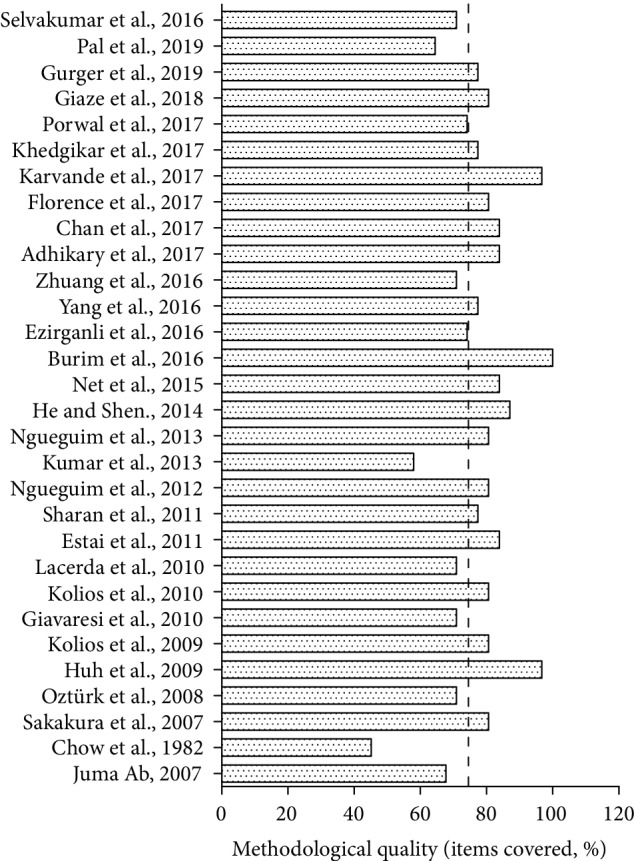
Analysis of methodological bias (reporting quality) for each study included in the review. Based on Animal Research: Reporting of In Vivo Experiments (ARRIVE) guidelines (http://www.nc3rs.org.uk/arrive-guidelines). The dotted line indicated the mean quality score (%). Detailed bias analysis stratified by domains and items evaluated is presented in Supplementary Material 1.

Detailed results of the bias analysis are depicted in Figure 3 and Table 4. An average of 77.63 ± 10.97 ARRIVE items were met by the original included studies. In general, the studies published more recently have better met the methodological quality criteria analyzed. Primary and secondary objectives were clearly stated by 77.42% (*n* = 24) of the studies, while 80.64% (*n* = 25) reported ethics committee permission for performing the research. The number of animals per group was reported in 83.87% (*n* = 26) of the studies, and only 32.26% (*n* = 10) reported a blind controlled study. Most studies provided information about the treatment description (90.32%, *n* = 28), the administered therapeutic dose (93.55%, *n* = 29), and treatment time (96.2%, *n* = 30). However, only 6.45% (*n* = 2) reported the period when the treatment was administered. All studies (100%, *n* = 31) reported the animal species, and 93.55% (*n* = 29) described the animal strain. The sex and weight were reported in 87.09% (*n* = 27) and 96.77% (*n* = 30) of the works, respectively, and 61.29% (*n* = 19) of the studies provided information about the animals' age. No study reported the description of genetic modification status, and 45.16% (*n* = 14) presented information regarding previous procedures performed on the animals. Among the articles, 29.03% (*n* = 9) reported the housing of experimental animals (facility type, cage or housing type, material, and number of cage companions), and 48.39% (*n* = 15) provided information about the experimental conditions (temperature, humidity, light cycles, feed, and water). Only 32.26% (*n* = 10) of the studies performed assessments and interventions related to animal welfare. Regarding the sample size, 74.19% (*n* = 23) of the studies reported the total number of animals used and the number of animals in each experimental group, but only 3.22% (*n* = 1) explained the reason for choosing such numbers. The details of how the animals were allocated to experimental groups (randomization or matching) were reported by 29.03% (*n* = 9) of the studies, and no study described the order in which animals in different groups were treated and assessed. The experimental outcomes were clear in 90.32% (*n* = 28) of the studies. Statistical analyses were performed by 90.32% (*n* = 28) of the studies, 87.09% (*n* = 27) of them specified the unit of analysis for each dataset, and 90.32% (*n* = 28) specified the methods used to assess whether the data met the assumptions of the statistical approach. Information regarding mortality was described in 6.45% (*n* = 2) of the studies, and no study described modifications to the experimental protocols made to reduce adverse events. A coherent interpretation of the results and the direct relationship between objectives and hypothesis were described in all included studies (100%, *n* = 31), and 19.35% (*n* = 6) commented on the limitations of the studies. Comments on the importance of applying the results to human biology were found in 45.16% (*n* = 14) of the studies, and 45.16% (*n* = 14) mentioned sources of funding and the role of the funder in the study.

## 4. Discussion

### 4.1. General Aspects

In this study, we conducted a systematic review to analyze the anti-inflammatory activity of plant extracts and their derivatives on bone repair in animal models. Despite the great heterogeneity of the studies, in general, the use of plant extracts was effective for treating bone lesions. We observed the release of markers and anti-inflammatory mediators after treatment with plants, which accelerated the recovery process of bone repair. In addition, histopathological and radiological analyses demonstrating bone remodeling (new bone formation, bone callus, cell proliferation, and osteogenesis) were the main findings of this study, which suggests that some components of the extracts may favor the proliferation of certain cell types. This occurs probably due to the interactions of these cells with the components of the extracts. Taking into consideration that the biological activity of a natural product is generally due to the synergism between its constituents, which potentiates its therapeutic properties, the study of plants for the treatment of many different diseases has been increasing gradually [51–53]. We believe that the development of therapeutic strategies based on the use of plants is opening a new perspective and represents a promising therapy as an alternative to conventional medicine and synthetic products [54–56].

The use of natural products for the treatment of injuries is an old practice [57] and represents an important source of bioactive compounds that contribute directly to the development of new drugs [58]. Our findings showed that most of the studies were conducted in China and India, countries known for having a millenary practice in traditional medicine [59]. This interest is probably due to the extensive and diverse flora found in these countries and to the vast traditional ethnomedicinal knowledge that serves as a basis for the researches [60, 61]. It is already known that the great ethnopharmacological potential of different phytotherapics favors and potentiates research in different health areas, thus directing the rational choice of medicinal plants [62, 63]. In addition, it is noteworthy that in China 40% of all health care provision is based on medicinal plants [64, 65]. However, the limiting factor found here was the language, which hinders access to information and reduces the dissemination of the data obtained to the scientific community [66].

As one of the objectives was to research experimental models closer to the human model, our study focused on *in vivo* experiments. Initially, all animal species were considered. However, after selection by inclusion criteria, only studies with rats, mice, and rabbits were admitted. It is noteworthy that there was a predominance of studies performed in murine models. Although these models do not allow the direct extrapolation of the results to human models [67], they can provide important insights into the biology and pathophysiology of the lesions and are indispensable for researchers [68]. The advantage of working with such animals is mainly due to reduced costs, as more animals can be housed in a limited space, and the shorter reproductive cycle. These characteristics allow, in a short time, a sufficient number of animals for large study groups, enabling a relevant statistical analysis [69].

### 4.2. Main Parameters Analyzed and Therapeutic Findings

The studies presented different methodologies, and this could be justified by the difference of objectives and parameters analyzed. However, important information such as sex, age, weight of the animals, and description of the methods for performing the induction of bone defect was neglected in some studies. The absence of this information compromises the comprehension of the studies, since biological and methodological variables directly affect the response to the treatments [70].

In addition to bone fracture, some studies have induced osteoporosis in animals, mainly to evaluate the action of phytotherapics as estrogen stimulants [25, 27, 29, 30, 37, 42]. Warriner et al. [71] published a systematic review investigating different works involving the association between bone fractures and osteoporosis and reported that the main fracture sites related to this disease were the vertebrae, femoral neck, radius, and ulna. In our review, we found that the most evaluated bone was the femur followed by the tibia, probably due to the greater resistance and size of these bones.

Two other parameters that varied widely were the size of the lesion or bone fracture and the treatment time, which can also compromise the reproduction of the work, as well as the comparison between the different groups treated with extracts. Image analysis, such as radiological findings and tomography, is fundamental for studies with fractures [72]. However, inflammatory and histopathological analyses play an important role in helping to interpret and confirm the cellular action of phytotherapeutic compounds in tissue repair [73]. The action of osteogenic cells on bone callus formation, cell organization, and the release of immunomarkers is a factor that can be confirmed by histology and immunohistochemistry [74–76], thus leading the experiment to a greater reliability of its results. The synthesis of proinflammatory mediators, in regions of spongy bone and compact bone, indicated in immunological and histopathological analyses, was higher in the phytotherapeutic treatment groups when compared to the control groups, demonstrating an efficiency of extracts in bone repair. This observation suggests that some of the components of the extract, or the synergism between them, may favor the synthesis of certain mediators and proliferation of cell types and, consequently, accelerate the synthesis of the bone matrix and the bone callus formation [77]. In this context, immunological, radiological, and histopathological analyses also confirm whether the fracture is strong and resistant and if it was totally, partially, or not closed. Similar results were found by Neto et al. [46] who evaluated the effect of a poultice prepared from the leaves of *Chenopodium ambrosioides L.* on bone repair in rabbits. Phytochemical analysis of the aqueous extract of this plant revealed the strong presence of saponins, flavonoids, tannins, and alkaloids, which may contribute to its effect on bone formation. These compounds are known for their anti-inflammatory and antioxidant action, accelerating the proliferation of anti-inflammatory proteins and enzymes such as catalase and superoxide dismutase that are responsible for protection during bone repair [46].

The use of phytotherapics has been increasing considerably in the last years, indicating that phytotherapy currently represents an effective way to treat the most varied tissue dysfunctions [78, 79]. This curative effect is probably related to the extensive source of bioactive compounds that are found in extracts obtained from natural products [80, 81]. However, the positive effects may vary according to the species of the plant and its used part. It is common to find studies that demonstrate, through different chromatographic analyses, different concentrations of flavonoids, tannins, and triterpenes in the bark and leaves of the same plant species [82–84], and that the protective effect of an extract should consider the part of the plant, possibly associated with its antioxidant effect [85]. In this review, we observed that approximately a quarter of the studies do not provide the scientific name of the species or the part of the plant used, which reduces the accuracy and reproducibility of these studies. The diversity of the compounds directly interferes with their performance in the organism, and their metabolites differ according to the parts of the plant, as well as the region and climate where they were collected [86]. As for the secondary metabolites found in the phytochemical description of the studies, we observed that 32.26% were flavonoids, indicating the positive action of this compound on the bone repair. The role of flavonoids in skin repair is already known, but recent studies have demonstrated the action of this compound also in bone repair [23, 29, 30, 36, 40, 41, 43, 45]. Another important fact to be informed in works with phytotherapics is the knowledge about the extraction techniques as well as the solvents used, since they can determine the isolation of a biologically active compound, directing the research [87]. This bias was found frequently in our study, since 19.35% of the studies did not report the solvent used for extraction which makes it difficult to obtain the extract again.

For the analysis of the work quality, we used an approach based on the ARRIVE guide, describing minimum information that can compromise the quality of the writing as well as the reproduction of the study [19]. Aspects related to the organization and writing of the evaluated articles showed that more than half of the studies presented an introduction with relevant scientific basis, as well as clearly written objectives. Through the bias analysis, flaws were detected in the reporting of the methodological procedures of the experiments, and it was found that many papers neglected information about the Ethics Committee approval, double-blind studies, and experimental conditions, such as light cycles and randomization. These results point out the need to improve experimental designs and current guidelines in reporting animal experiments as means to ensure an adequate level of scientific evidence [88].

### 4.3. Limitations of the Study

A great contribution of this work is based on the global estimation of the use of plant extracts for the treatment of bone lesions. However, the results presented here should be interpreted with caution, since it can be argued that the selection process of the studies may be biased due to different factors, such as the initial exclusion based only on the reading of the titles and abstracts or the inclusion of more than one study of the same group of researchers. However, the work selection process carried out in our review was widely based on recommended and accepted practices for performing systematic reviews [17, 89].

Another relevant issue highlighted in our work is related to the bias of the publication. After this analysis, we realized that aspects related to the organization of the experiments were neglected, including lack of randomization. These factors highlight the need to enhance experimental designs and current guidelines in reporting animal experiments as means to ensure an adequate level of scientific evidence. Although most studies indicate possible effects such as osteoblastic proliferation, angiogenesis, and increased formation of the bone matrix with fracture closure and bone callus formation, the mechanisms of action of extracts on bone tissue are unclear. In addition, the lack of phytochemical characterization of most plant extracts makes it impossible to identify the compounds responsible for the positive effect.

Finally, it was observed that the methodologies used and the evaluation parameters are extremely heterogeneous, with different measures being reported in all the studies, such as analysis of the size of the lesion or fracture, as well as histopathological analyses. Despite the improvement in the methodological quality of individual studies from 2009, much still needs to be done to allow the reproducibility of the studies. Interestingly, most papers did not report whether the results of their studies could be translated into other species and systems, including some relevance to human biology [90]. Considering the experimental model used in most studies and the social relevance of bone lesions for the world population, the translation of the results and their applicability on the treatment of human diseases are fundamental to allow the continuity of studies with medicinal plants, once the goal is to develop a drug that improves the human quality of life.

## 5. Conclusion

The results of this study demonstrated that the use of plant extracts stimulates bone repair, increasing osteogenesis, the rate of calcification, and the formation and mineralization of bone callus, accelerating the process of new bone formation on the fracture region. Possibly, these effects are related to anti-inflammatory and antioxidant power of these extracts. However, the methodological flaws found in some studies make it difficult to understand and use data in studies for the human condition. Therefore, more complete methodological descriptions are needed to better compare the studies and to allow the reproducibility of future trials.

## Figures and Tables

**Table 1:** Description of the main characteristics of the animal model in studies demonstrating the action of plant extracts in the bone repair process.

Title	Study ID	Country	Animal model	Sex (M/F)∗	Age (d, w, and m)∗	Weight
The effect of *Davallina orientalis* on bone healing-a preliminary report	Chow et al., 1982 [20]	China	Mice	?	11-12 w	28-33 g
The effects of *Lepidium sativum* seeds on fracture-induced healing in rabbits	Juma Ab, 2007 [21]	Saudi Arabia	New Zealand white rabbits	?	6 m	4-5 kg
Influence of homeopathic treatment with comfrey on bone density around titanium implants. A digital subtraction radiography study in rats	Sakakura et al., 2007 [22]	Brazil	Wistar rats	M	2 m	180–220 g
The effects of phytoestrogens on fracture healing: experimental research in New Zealand white rabbits	Oztürk et al., 2008 [23]	Turkey	New Zealand white rabbits	?	?	1.62 ± 0.05 kg
Formononetin promotes early fracture healing through stimulating angiogenesis by up-regulating VEGFR-2/Flk-1 in a rat fracture model	Huh et al., 2009 [24]	Korea	Sprague-Dawley rats	M	2 m	280-300 g
Equol but not genistein improves early metaphyseal fracture healing in osteoporotic rats	Kolios et al., 2009 [25]	Germany	Sprague-Dawley rats	F	3 m	?
Bone regeneration potential of a soybean-based filler: experimental study in a rabbit cancellous bone defects	Giavaresi et al., 2010 [26]	Italy	New Zealand white rabbits	M	Adult	3.250 ± 0.350 kg
Absence of positive effect of black cohosh (*Cimicifuga racemosa*) on fracture healing in osteopenic rodent model	Kolios et al., 2010 [27]	Germany	Sprague-Dawley rats	F	3 m	?
Bone quality associated with daily intake of coffee: a biochemical, radiographic and histometric study	Lacerda et al., 2010 [28]	Brazil	Wistar rats	?	?	250-300 g
*Piper sarmentosum* enhances fracture healing in ovariectomized osteoporotic rats: a radiological study	Estai et al., 2011 [29]	Malaysia	Sprague-Dawley rats	F	?	200-250 g
A novel quercetin analogue from a medicinal plant promotes peak bone mass achievement and bone healing after injury and exerts an anabolic effect on osteoporotic bone: the role of aryl hydrocarbon receptor as a mediator of osteogenic action	Sharan et al., 2011 [30]	India	Sprague-Dawley rats	F	?	200 ± 20 g
Evaluation of Cameroonian plants towards experimental bone regeneration	Ngueguim et al., 2012 [31]	Cameroon	Sprague-Dawley rats	F	4 m	220 ± 20 g
The bone fracture-healing potential of *Ormocarpum cochinchinense*, methanolic extract on albino Wistar rats	Kumar et al., 2013 [32]	India	Wistar albino rats	F	3 m	150-200 g
Ethanol extract of *Peperomia pellucida* (Piperaceae) promotes fracture healing by an anabolic effect on osteoblasts	Ngueguim et al., 2013 [33]	Cameroon	Sprague-Dawley rats	F	4 m	200 ± 20 g
Salvianolic acid B promotes bone formation by increasing activity of alkaline phosphatase in a rat tibia fracture model: a pilot study	He and Shen, 2014 [34]	China	Sprague-Dawley rats	M	7 w	225 g
*Chenopodium ambroisioides* in the repair of fractures in rabbits	Neto et al., 2015 [35]	Brazil	New Zealand white rabbits	M	Adult	3.0 ± 0.5 kg
Repair of critical calvarias defects with systemic *Epimedium sagittatum* extract	Burim et al., 2016 [36]	Brazil	Wistar albino rats	M	?	200 − 250 g
The effects of *Nigella sativa* seed extract on bone healing in an experimental model	Ezirganli et al., 2016 [37]	Turkey	Wistar albino rats	F	3 m	280-310 g
Excavating the role of *Aloe Vera* wrapped mesoporous hydroxyapatite frame ornamentation in newly architecture polyurethane scaffolds for osteogenesis and guided bone regeneration with microbial protection	Selvakumar et al., 2016 [38]	India	New Zealand white rabbits	M	?	2 kg
Root bark of *Sambucus williamsii* Hance promotes rat femoral fracture healing by the BMP-2/Runx2 signaling pathway	Yang et al., 2016 [39]	China	Sprague-Dawley rats	M/F	3 m	220 ± 20 g
*Ulmus davidiana* extract improves lumbar vertebral parameters in ovariectomized osteopenic rats	Zhuang et al., 2016 [40]	China	Rats	F	?	250-270 g
Dried and free flowing granules of *Spinacia oleracea* accelerate bone regeneration and alleviate postmenopausal osteoporosis	Adhikary et al., 2017 [41]	India	Sprague-Dawley rats	F	3 m	180-200 g
Tanshinol alleviates osteoporosis and myopathy in glucocorticoid-treated rats	Chen et al., 2017 [42]	China	Sprague-Dawley rats	F	4-5 m	250-275 g
Aqueous extract of *Peperomia pellucida* (L.) HBK accelerates fracture healing in Wistar rats	Florence et al., 2017 [43]	Cameroon	Wistar rats	F	3 m	150-200 g
Heartwood extract from *Dalbergia sissoo* promotes fracture healing and its application in ovariectomy-induced osteoporotic rats	Karvande et al., 2017 [44]	India	Sprague-Dawley rats	F	?	220 ± 20 g
Ethanolic extract of *Dalbergia sissoo* promotes rapid regeneration of cortical bone in drill-hole defect model of rat	Khedgikar et al., 2017 [45]	India	Sprague-Dawley rats	F	?	180 ± 20 g
*Chenopodium ambrosioides* as a bone graft substitute in rabbits radius fracture	Neto et al., 2017 [46]	Brazil	New Zealand white rabbits	M	Adult	3.0 ± 0.5 kg
Guava fruit extract and its triterpene constituents have osteoanabolic effect: stimulation of osteoblast differentiation by activation of mitochondrial respiration via the Wnt/*β*-catenin signaling	Porwal et al., 2017 [47]	India	Sprague-Dawley rats	F	?	220 ± 20 g
*Marantodes pumilum* leaves promote repair of osteoporotic fracture in postmenopausal Sprague-Dawley rats	Giaze et al., 2018 [48]	Malaysia	Sprague-Dawley rats	F	4 m	250-300 g
Grape seed extract supplement increases bone callus formation and mechanical strength: an animal study	Gurger et al., 2019 [49]	Turkey	Wistar-Albino	M	2-3 m	350 ± 50 g
Extract and fraction of *Cassia occidentalis* L. (a synonym of *Senna occidentalis*) have osteogenic effect and prevent glucocorticoid-induced osteopenia	Pal et al., 2019 [50]	India	Sprague-Dawley rats	M	2-3 m	220 ± 20 g

M: male; F: female; d: day; w: week; m: month; ?: not related.

**Table 2:** Description of the main characteristics of the treatment in studies demonstrating the action of plant extracts in the bone repair process.

Study ID	Number of animals/group	Control group	Treatment time (d, w, and m)∗	Osteoporosis	Bone type	Induced defect	Lesion size	Anesthesia (pharmaco)	Postoperative drug	Euthanasia
Chow et al., 1982 [20]	10	Saline	?	No	Femur	Osteotomy	?	Ether inhalation	?	?
Juma Ab, 2007 [21]	3	Routine diet	12 w	No	Femur	Drill machine	?	Ketamine/xylazine	?	?
Sakakura et al., 2007 [22]	24	Not receiving treatment	28 d	No	Tibia	Rotary drills	2 cm	Ketamine/xylazine	Pentabiótico	?
Oztürk et al., 2008 [23]	11	?	35 d	No	Tibia	?	?	Alfazin/propofol	?	?
Huh et al., 2009 [24]	144/12	Saline	21 d	No	Femur	?	?	?	?	Paraformaldehyde/tiletamine-zolazepam
Kolios et al., 2009 [25]	12	Phytoestrogen-free	35 d	Yes OVX	Tibia	Osteotomy	?	Ketamine/xylazine	?	Decapitated under deep CO_2_ anesthesia
Giavaresi et al., 2010 [26]	2/18/4	Not receiving treatment	24 w	No	Femur	Drill machine	6 mm	?	Enfloroxacin (100 mg) and metamizole chloride (50 mg/kg)	General anesthesia (Tanax)
Kolios et al., 2010 [27]	12	Phytoestrogen-free	35 d	Yes OVX	Tibia	Osteotomy	?	Ketamine/xylazine	?	Decapitated under deep CO_2_ anesthesia
Lacerda et al., 2010 [28]	?	Water	7, 21, 42 d	No	Maxilla	Incisor tooth extraction	?	2,2,2-Tribromoethanol	Pentabiotic	Anesthetic overdose
Estai et al., 2011 [29]	6	Saline	6 w	Yes OVX	Femur	Guillotine	?	Xylazil/ketamine	Antibiotic Baytril and povidone-iodine solution	?
Sharan et al., 2011 [30]	10	Acacia gum in water	2 w	Yes OVX	Skull	Drill machine	0.8 mm diameter	?	?	?
Ngueguim et al., 2012 [31]	6	Acacia gum in water	12 d	No	Femur	Drill machine	0.8 mm diameter	?	?	?
Kumar et al., 2013 [32]	3	Saline	0, 7, 14, 21 d	No	Femur	Fracture device	?	Ketamine/hydrochloride	?	?
Ngueguim et al., 2013 [33]	6	Acacia gum in water	12 d	No	Femur	Drill machine	0.8 mm diameter	?	?	?
He and Shen, 2014 [34]	10	Saline	12 w	No	Tibia	Custom-made three-point	?	?	?	High-dose ketamine
Neto et al., 2015 [35]	10	0.9% NaCl	10 d	No	Radius	Oscillating bone saw	1 cm	Ketamine/xylazine	?	Anesthetic overdose
Burim et al., 2016 [36]	20	Saline	7, 14, 21, 42 d	No	Calvaria	Steel trephine drill	8 mm diameter	Ketamine/xylazine/hydrochloride	Antibiotic prophylaxis (benzathine benzylpenicillin)	CO_2_ chamber
Ezirganli et al., 2016 [37]	16	?	2, 4 w	Yes OVX	Calvaria	Trephine burr	5 mm diameter	Ketamine/xylazine	?	Barbiturate overdose
Selvakumar et al., 2016 [38]	?	?	4 w	No	Tibia	Drill machine	4 mm	Ketamine/xylazine	Meloxicam oral suspension (0.2 mg/kg) and enrofloxacin (5 mg/kg)	?
Yang et al., 2016 [39]	24	Water	2, 4, 8 w	No	Femur	Osteotomy	?	Chloral hydrate	Penicillin sodium	?
Zhuang et al., 2016 [40]	8	Methylcellulose	14 d	No	Femur	Drill machine	0.8 mm diameter	Ketamine/xylazine	?	?
Adhikary et al., 2017 [41]	10	Parathyroid hormone (PTH)	14 d	?	Femur	Drill machine	0.8 mm diameter	?	?	?
Chen et al., 2017 [42]	24 or 16	Water/calcitriol	6 w	Yes GIOP	Tibia	?	2 mm hole	Chloral hydrate	?	Cardiac puncture
Florence et al., 2017 [43]	5	Water	14 d	No	Femur	Drill machine	?	?	?	Decapitated under ketamine/valium anesthesia
Karvande et al., 2017 [44]	10	Acacia gum in water	2 w	No	Femur	Drill machine	0.8 mm diameter	Ketamine/xylazine	?	?
Khedgikar et al., 2017 [45]	?	Acacia gum in water	2 w	No	Femur	Drill machine	0.8 mm diameter	?	?	?
Neto et al., 2017 [46]	12	Not receiving treatment	10 d	No	Radius	Oscillating bone saw	1 cm	Ketamine/xylazine	?	Lethal doses of the anesthetics
Porwal et al., 2017 [47]	?	Water	12 d	No	Femur	Drill machine	0.8 mm diameter	Ketamine/xylazine	?	?
Giaze et al., 2018 [48]	6	Estrogen (Premarin®)	4 m	Yes	Tibia	Pulse ultrasound	0.5 mm	Ketamine/xylazine	Enrofloxacin (Baytril®) 5 mg/kg and buprenorphine 0.1 mg/kg	Ketamine-xylazine mixture overdose and cervical dislocation
Gurger et al., 2019 [49]	8	Standard diet	10, 20, and 30 d	No	Femur	Multidrilling technique	0.5cm	Ketamine/xylazine	Buprenorphine (0.05 mg/kg) and cefazolin (30 mg/kg)	Sodium pentobarbital (400 mg/kg)
Pal et al., 2019 [50]	10	Water	14 w	Yes GIO	Femur	Drill machine	0.8 mm	Ketamine/xylazine	?	Ketamine/xylazine overdose

d: day; w: week; m: month; OVX: ovariectomized; GIOP: Glucocorticoid-Induced Osteoporosis Program; GIO: GC-induced osteoporosis; ?: not related.

**Table 3:** Main characteristics of the plant, extraction form, route, and dose administered in studies demonstrating the action of plant extracts in the bone repair process.

Study ID	Plant	Used parts	Indication	Solvent used for extraction	Obtaining plant material	Dose	Administration	Secondary metabolites	Geographic distribution
Selvakumar et al., 2016 [38]	*Aloe vera*	Leaves	Anti-inflammatory, antioxidant activity, immune modulatory, and burn wounds	Water	?	?	Grafts	Saponins	?
Huh et al., 2009 [24]	*Astragalus membranaceus*	Root	Vascular diseases, breast cancer, climacteric bone diseases (reports)	Water/ethanol	Seoul, Korea	20 *μ*g/kg/day and 200 *μ*g/kg/day	Orally, *ad libitum*	Isoflavone formononetin	?
Pal et al., 2019 [50]	*Cassia occidentalis*	Stem and leaves	Purgative, febrifuge, diuretic, and treatment of fracture and bone diseases	Ethanol	Lucknow, India	250 mg/kg and 100 mg/kg	Orally, *ad libitum*	Flavonoids	South Asia and South America
Neto et al., 2015 [35]	*Chenopodium ambroisioides*	Leaves	Contusions and fractures	Water	Brazil	10 mL	Topical	?	Brazil and Latin America
Neto et al., 2017 [46]	*Chenopodium ambroisioides*	Leaves	Inflammatory conditions, contusions, and fractures	Water	Brazil	20 g	Grafts	Flavonoids, alkaloids, and saponins	Brazil and Latin America
Kolios et al., 2010 [27]	*Cimicifuga racemosa*	Rhizomes	Reduce climacteric complaints (proven)	Water/ethanol	?	24.9 mg/day	Orally, *ad libitum*	?	?
Lacerda et al., 2010 [28]	Coffee	?	Protein expression of the vitamin D receptor, osteoblast activity, anti-inflammatory (reports)	Water	SP, Brazil	50 mg/mL	Orally, *ad libitum*	Caffeine (reports)	?
Sakakura et al., 2007 [22]	Comfrey (*Shymphytum officinalis*)	?	Fractured bone, tendon damage, joint disease, and ulcerations in the gastrointestinal tract	Ethanol	?	6CH (homeopathic dose)	Grafts	?	?
Karvande et al., 2017 [44]	*Dalbergia sissoo*	Heartwood	Stimulation of new cell growth, tissue regeneration (reports) Fevers, anti-inflammation (traditional uses)	Ethanol	Lucknow, India	250, 500, and 1000 mg/kg/day	?	Neoflavonoids (reports)	Indian subcontinent
Khedgikar et al., 2017 [45]	*Dalbergia sissoo*	Leaves	Stimulation of new bone cells, tissue regeneration, anti-inflammatory (reports)	Ethanol	Lucknow, India	250, 500, and 1000 mg/kg/day	Orally, *ad libitum*	Phytoestrogens, flavonoids (reports)	Indian subcontinent
Chow et al., 1982 [20]	*Davallina orientalis*	?	Fractures (traditional uses)	Methanol	?	10 mg/kg or 30 mg/kg	Intraperitoneal	?	?
Ngueguim et al., 2012 [31]	*Elephantopus mollis*, *Spilanthes africana*, *Urena lobata*, *Momordica multiflora*, *Asystasia gangetica*, and *Brillantaisia ovariensis*	Leaves, twigs, or whole plant	Bone diseases and fracture repair, anti-inflammatory (traditional uses)	Ethanol	Dschang region, Cameroon	250, 500, and 750 mg/kg	Orally, *ad libitum*	?	?
Burim et al., 2016 [36]	*Epimedium sagittatum*	Dried leaves	Bone repair, osteoporosis, anti-inflammatory (reports)	Water/ethanol	Shaanxi, China	0.3 mL	Gavage	Flavonoid icariin	Asian countries
Gurger et al., 2019 [49]	Grape seed	Seed	Vasodilator, antiallergic, immunostimulator, anti-inflammatory, cardioprotective, antiviral, antibacterial, and anticarcinogen activities	1% carboxymethyl cellulose	United States of America	100 mg/kg/day	Gavage	?	?
Juma Ab, 2007 [21]	*Lepidium sativum*	Seeds	Diuresis, bile function, cough, fracture healing, anti-inflammatory (traditional uses)	?	Saudi Arabia	6 g/day	Orally, *ad libitum*	?	?
Giaze et al., 2018 [48]	*Marantodes pumilum* var. *alata*	Leaves and roots	Reproductive health problems and postmenopausal symptoms, known to protect the bone against osteoporosis	Water	Malaysia	20 and 100 mg/kg/day	Orally, *ad libitum*	Phenolic compounds	?
Ezirganli et al., 2016 [37]	*Nigella sativa*	Seed	Analgesic, antipyretic, anti-inflammatory, antimicrobial, antibacterial, antifungal, antiparasitic, antiasthmatic, antioxidant, antineoplastic	?	?	10 mg/kg/day	Gavage	Proteins, alkaloids, essential oils, saponin	?
Kumar et al., 2013 [32]	*Ormocarpum cochinchinense*	Leaves	Fractures (traditional uses)	Methanol	Kancheepuram district, Tamil Nadu, India	100 mg/kg^−1^	Topical/orally, *ad libitum*	?	Jungles of the Coromandel coast and dry forest from Tamil Nadu, India
Ngueguim et al., 2013 [33]	*Peperomia pellucida* (L.) HBK	Whole plant	Fractures, abdominal pain, headache, hypertension, anti-inflammatory (traditional uses)	Ethanol	Dschang region, Cameroon	100 and 200 mg/kg	Orally, *ad libitum*	?	Damp areas from Cameroon
Florence et al., 2017 [43]	*Peperomia pellucida* (L.) HBK	Whole plant	Abdominal pain, anti-inflammatory, boils, colic, fatigue, gout, rheumatic, joint pain, fracture management (traditional uses)	Water	Limbe, Cameroon	100, 200, and 400 mg/kg	Orally, *ad libitum*	Flavonoids (reports)	America, Africa, and Asia
Estai et al., 2011 [29]	*Piper sarmentosum*	Leaves	Diabetes, hypertension, and joint aches (traditional uses)	?	?	125 mg/kg/day	Orally, *ad libitum*	Alkaloids, amides, flavonoids, lignans, phenylpropanoids (reports)	?
Porwal et al., 2017 [47]	*Psidium guajava*	Fruits	Diabetes, obesity, osteoporosis (traditional uses)	Ethanol	Sitapur, Uttar Pradesh, India	250 mg/kg	?	Polyphenols, carotenoids (reports)	?
Chen et al., 2017 [42]	*Salvia miltiorrhiza*	?	Osteoporosis, osteogenesis, anti-inflammatory (reports)	?	?	25 and 50 mg/kg	Gavage	Tanshinol	?
Yang et al., 2016 [39]	*Sambucus williamsii* Hance (SWH)	Root bark	Fractures, anti-inflammatory, osteoporosis (traditional uses)	Ethanol	Harbin, China	340 and 680 mg/kg	Orally, *ad libitum*	Lignans, iridoids	China
Kolios et al., 2009 [25]	Soybeans	?	Osteoporosis (reports)	?	?	1 g/kg	Orally, *ad libitum*	Isoflavone genistein	?
Giavaresi et al., 2010 [26]	Soybean	?	?	Water/ethanol	?	?	Grafts	Isoflavones, phytoestrogens	?
Adhikary et al., 2017 [41]	*Spinacia oleracea*	Whole plant	Increased satiety in females and lipid-lowering effects in postmenopausal women (previous reports)	Ethanol	?	125, 250, 500, and 750 mg/kg/day	Orally, *ad libitum*	Ascorbate, carotenoids, tocopherols, phenolics, flavonoids, folate	?
Zhuang et al., 2016 [40]	*Ulmus davidiana* Planch	Stem bark	Anti-inflammation, edema, stomach cancer (traditional uses)	Ethanol	?	50, 100, 250, and 500 mg/kg	Gavage	Flavonoids (catechin)	Korean Peninsula
Sharan et al., 2011 [30]	*Ulmus wallichiana*	Stem bark	Fractures (traditional uses)	Ethanol	?	1.0 mg/kg/day and 5.0 mg/kg/day	Orally, *ad libitum*	Flavonoid quercetin	?
Oztürk et al., 2008 [23]	*Vitex agnus-castus* L.	Fruits	Bone loss and resorption, heart disease (reports)	Ethanol	?	0.75 mg	Intramuscular	Flavonoids (reports)	Middle East and Southern Europe

?: not related; CH: diluted 100×.

**Table 4:** Analysis of methodological bias of the studies demonstrating the action of plant extracts in the bone repair process.

	Chow et al., 1982 [20]	Juma Ab, 2007 [21]	Sakakura et al., 2007 [22]	Oztürk et al., 2008 [23]	Huh et al., 2009 [24]	Kolios et al., 2009 [25]	Giavaresi et al., 2010 [26]	Kolios et al., 2010 [27]	Lacerda et al., 2010 [28]	Estai et al., 2011 [29]	Sharan et al., 2011 [30]	Ngueguim et al., 2012 [31]	Kumar et al., 2013 [32]	Ngueguim et al., 2013 [33]	He and Shen, 2014 [34]	Neto et al., 2015 [35]	Burim et al., 2016 [36]	Ezirganli et al., 2016 [37]	Selvakumar et al., 2016 [38]	Yang et al., 2016 [39]	Zhuang et al., 2016 [40]	Adhikary et al., 2017 [41]	Chen et al., 2017 [42]	Florence et al., 2017 [43]	Karvande et al., 2017 [44]	Khedgikar et al., 2017 [45]	Neto et al., 2017 [46]	Porwal et al., 2017 [47]	Giaze et al., 2018 [48]	Gurger et al., 2019 [49]	Pal et al., 2019 [50]		
Title																																	
Accurate and concise description of the article content	X	X	X	X	X	X	X	X	X	X	X		X	X	X	X	X	X	X	X	X	X		X	X	X	X	X	X	X	X	29	93.55%

Abstract																																	
Background summary, research objectives, methods, principal findings, and conclusions		X	X	X	X	X	X	X	X	X	X	X		X	X	X	X		X	X			X	X		X	X		X	X	X	24	77.42%

Introduction																																	
Sufficient scientific background		X	X	X	X	X	X	X	X	X	X	X	X	X	X	X	X	X	X	X	X	X	X	X	X	X	X	X	X	X	X	30	96.77%
Explanation of the experimental approach and rationale	X	X	X	X	X	X	X	X	X	X	X	X	X	X	X	X	X	X	X	X	X	X	X	X	X	X	X	X	X	X	X	31	100%

Objectives																																	
Clear primary and second objectives	X	X	X	X	X	X	X	X	X	X	X	X		X	X	X	X		X		X	X			X	X	X	X		X		24	77.42%

Materials and methods																																	
Nature of the ethical review permissions, relevant licenses, and national or institutional guidelines for the care and use of animals					X		X	X	X	X	X	X	X	X	X	X	X	X	X	X	X	X	X	X	X	X	X	X	X		X	25	80.64%
*Study design*																																	
Number of animals per group	X	X	X	X	X	X	X	X		X	X	X	X	X	X	X	X	X		X	X	X	X	X	X		X		X	X		26	83.87%
Information on whether the experiment was performed as a blind controlled study			X				X	X				X		X		X	X								X		X				X	10	32.26%
*Experimental procedures*																																	
Treatment description	X	X	X	X	X	X	X	X	X		X	X	X	X	X	X	X		X	X	X	X	X	X	X	X		X	X	X	X	28	90.32%
Treatment dosage	X	X	X	X	X	X		X	X	X	X	X	X	X	X	X	X	X	X	X	X	X	X	X	X	X		X	X	X	X	29	93.55%
Treatment duration		X	X	X	X	X	X	X	X	X	X	X	X	X	X	X	X	X	X	X	X	X	X	X	X	X	X	X	X	X	X	30	96.77%
Time of day of treatment administration																	X						X									2	6.45%
*Experimental animals*																																	
Information regarding animal species	X	X	X	X	X	X	X	X	X	X	X	X	X	X	X	X	X	X	X	X	X	X	X	X	X	X	X	X	X	X	X	31	100%
Animals' strain		X	X	X	X	X	X	X	X	X	X	X	X	X	X	X	X	X	X	X		X	X	X	X	X	X	X	X	X	X	29	93.55%
Animals' sex			X		X	X	X	X		X	X	X	X	X	X	X	X	X	X	X	X	X	X	X	X	X	X	X	X	X	X	27	87.09%
Animals' weight range	X	X	X	X	X	X	X		X	X	X	X	X	X	X	X	X	X	X	X	X	X	X	X	X	X	X	X	X	X	X	30	96.77%
Animals' age	X	X	X		X	X	X	X			X	X	X	X	X			X	X	X		X	X	X					X			19	61.29%
Description of genetic modification status (knockout, transgenic, SPF)																																0	0%
Information related to previous procedures performed on the animals								X	X	X			X			X		X		X		X	X	X	X			X	X	X		14	45.16%
*Housing and husbandry*																																	
Housing of experimental animals (facility type, cage or housing type, material, number of cage companions)		X	X		X			X	X				X				X								X				X			9	29.03%
Husbandry conditions (breeding program, light/dark cycle, temperature, water)								X	X	X			X			X	X	X			X	X	X	X	X	X		X		X		15	48.39%
Welfare-related assessments and interventions that were carried out before, during, or after the experiment			X							X			X				X	X		X			X				X		X	X		10	32.26%
*Sample size*																																	
Total number of animals used in each experimental group and the number of animals in each experimental group	X	X	X	X	X	X		X		X	X	X			X	X	X	X	X	X	X		X	X	X		X		X	X		23	74.19%
Explanation regarding the decision of the number of animals and details of sample size calculation					X																											1	3.22%
*Allocating animals to experimental groups*																																	
Full details of how animals were allocated to experimental groups (including randomization or matching)				X						X					X	X	X					X	X			X	X					9	29.03%
Order in which the animals in the different experimental groups were treated and assessed																																0	0%
*Experimental outcomes*																																	
Clear experimental outcomes assessed	X	X	X	X	X	X	X	X	X			X		X	X	X	X	X	X	X	X	X	X	X	X	X	X	X	X	X	X	28	90.32%
*Statistical methods*																																	
Statistical methods used for each analysis		X	X	X	X	X	X		X	X	X	X		X	X	X	X	X	X	X	X	X	X	X	X	X	X	X	X	X	X	28	90.32%
Unit of analysis specifications for each dataset			X	X	X	X	X		X	X	X	X		X	X	X	X	X	X	X	X	X	X	X	X	X	X	X	X	X	X	27	87.09%
Methods used to assess whether the data met the assumptions of the statistical approach		X	X	X	X	X	X		X	X	X	X		X	X	X	X	X	X	X	X	X	X	X	X	X	X	X	X	X	X	28	90.32%

Results																																	
*Baseline data*																																	
Description of animals' health status, for each experimental group, before treatment																																0	0%
*Number analyzed*																																	
Number of animals in each group included in each analysis (absolute numbers)	X	X	X	X	X	X	X	X	X	X	X	X		X	X	X	X	X		X	X	X	X	X	X	X	X	X	X	X	X	29	93.55%
Animals or data not included in the analysis (and explanation for the exclusion)					X			X																								2	6.45%
*Outcomes and estimation*																																	
Information (mean = standard deviation)	X		X	X	X	X	X	X	X	X	X	X		X	X	X	X		X	X	X	X	X	X	X	X	X	X	X	X	X	28	90.32%
*Adverse events*																																	
Information regarding mortality of experimental animals (mean = standard deviation)					X										X																	2	6.45%
Modifications to the experimental protocols made to reduce adverse events																																0	0%

Discussion																																	
*Interpretation/scientific implications*																																	
Interpretation of the results, taking into account the study objectives and hypotheses, current theory, and relevant studies	X	X	X	X	X	X	X	X	X	X	X	X	X	X	X	X	X	X	X	X	X	X	X	X	X	X	X	X	X	X	X	31	100%
Comments on the study limitations (sources of bias, limitations of the animal model, imprecision associated with the results)					X	X		X			X				X		X															6	19.35%
*Generalisability/translation*																																	
Comments on how the findings are likely to translate to other species or systems, including relevance to human biology		X			X	X				X		X		X			X	X	X		X	X			X	X		X				14	45.16%
*Funding*																																	
List of funding sources and the role of the funder(s) in the study				X	X	X				X	X	X		X	X		X					X		X	X	X		X				14	45.16%
Total results	14	21	25	22	30	25	22	25	22	26	24	25	18	25	27	26	31	23	22	24	22	26	26	25	30	24	23	23	25	24	20		

X: related; unmarked: not related.

## Supplementary Materials

Supplementary Materials Table S1: full search strategy in PubMed and Scopus, including search terms and filters.
