# Supplementary material for: Plant Extracts in the Bone Repair Process: A Systematic Review
Source: Mediators Inflamm. 2019 Nov 25;2019:1296153. doi: 10.1155/2019/1296153 (PMC6899290; doi:10.1155/2019/1296153)
Supplement: Supplementary Materials — Table S1: full search strategy in PubMed and Scopus, including search terms and filters. [file 1296153.f1.pdf]

Table S1: Full search strategy in PubMed and Scopus, including search terms and filters.

Data base	Descriptors	Items Found	Time	Date
PubMed	Animals #1 OR #2<br><br>#1 Filters<br><br>("animal experimentation"[MeSH Terms] OR "models, animal"[MeSH Terms] OR "invertebrates"[MeSH Terms] OR "Animals"[Mesh:noexp] OR "animal population groups"[MeSH Terms] OR "chordata"[MeSH Terms:noexp] OR "chordata, nonvertebrate"[MeSH Terms] OR "vertebrates"[MeSH Terms:noexp] OR "amphibians"[MeSH Terms] OR "birds"[MeSH Terms] OR "fishes"[MeSH Terms] OR "reptiles"[MeSH Terms] OR "mammals"[MeSH Terms:noexp] OR "primates"[MeSH Terms:noexp] OR "artiodactyla"[MeSH Terms] OR "carnivora"[MeSH Terms] OR "cetacea"[MeSH Terms] OR "chiroptera"[MeSH Terms] OR "elephants"[MeSH Terms] OR "hyraxes"[MeSH Terms] OR "insectivora"[MeSH Terms] OR "lagomorpha"[MeSH Terms] OR "marsupialia"[MeSH Terms] OR "monotremata"[MeSH Terms] OR "perissodactyla"[MeSH Terms] OR "rodentia"[MeSH Terms] OR "scandentia"[MeSH Terms] OR "sirenia"[MeSH Terms] OR "xenarthra"[MeSH Terms] OR "haplorhini"[MeSH Terms:noexp] OR "strepsirhini"[MeSH Terms] OR "platyrrhini"[MeSH Terms] OR "tarsii"[MeSH Terms] OR "catarrhini"[MeSH Terms:noexp] OR "cercopithecidae"[MeSH Terms] OR "hylobatidae"[MeSH Terms] OR "hominidae"[MeSH Terms:noexp] OR "gorilla gorilla"[MeSH Terms] OR "pan paniscus"[MeSH Terms] OR "pan troglodytes"[MeSH Terms] OR "pongo pygmaeus"[MeSH Terms])<br><br>#2 Filters<br><br>((animals[TIAB] OR animal[TIAB] OR mice[TIAB] OR mus[TIAB] OR mouse[TIAB] OR murine[TIAB] OR woodmouse[TIAB] OR rats[TIAB] OR rat[TIAB] OR murinae[TIAB] OR muridae[TIAB] OR cottonrat[TIAB] OR cottonrats[TIAB] OR hamster[TIAB] OR hamsters[TIAB] OR cricetinae[TIAB] OR rodentia[TIAB] OR rodent[TIAB] OR rodents[TIAB] OR pigs[TIAB] OR pig[TIAB] OR swine[TIAB] OR swines[TIAB] OR piglets[TIAB] OR piglet[TIAB] OR boar[TIAB] OR boars[TIAB] OR "sus scrofa"[TIAB] OR ferrets[TIAB] OR ferret[TIAB] OR polecat[TIAB] OR polecats[TIAB] OR "mustela putorius"[TIAB] OR "guinea pigs"[TIAB] OR "guinea pig"[TIAB] OR cavia[TIAB] OR callithrix[TIAB] OR marmoset[TIAB] OR marmosets[TIAB] OR cebuella[TIAB] OR hapale[TIAB] OR octodon[TIAB] OR chinchilla[TIAB] OR chinchillas[TIAB] OR gerbillinae[TIAB] OR gerbil[TIAB] OR gerbils[TIAB] OR jird[TIAB] OR jirds[TIAB] OR merione[TIAB] OR meriones[TIAB] OR rabbits[TIAB] OR rabbit[TIAB] OR hares[TIAB] OR hare[TIAB] OR diptera[TIAB] OR flies[TIAB] OR fly[TIAB] OR dipteral[TIAB] OR drosphila[TIAB] OR drosophilidae[TIAB] OR cats[TIAB] OR cat[TIAB] OR carus[TIAB] OR felis[TIAB] OR nematoda[TIAB] OR nematode[TIAB] OR nematoda[TIAB] OR nematode[TIAB] OR nematodes[TIAB] OR sipunculida[TIAB] OR dogs[TIAB] OR dog[TIAB] OR canine[TIAB] OR canines[TIAB] OR canis[TIAB] OR sheep[TIAB] OR sheeps[TIAB] OR mouflon[TIAB] OR mouflons[TIAB] OR ovis[TIAB] OR goats[TIAB] OR goat[TIAB] OR capra[TIAB] OR capras[TIAB] OR rupicapra[TIAB] OR chamois[TIAB] OR haplorhini[TIAB] OR monkey[TIAB] OR monkeys[TIAB] OR anthropoidea[TIAB] OR anthropoids[TIAB] OR saguinus[TIAB] OR tamarin[TIAB] OR tamarins[TIAB] OR leontopithecus[TIAB] OR hominidae[TIAB] OR ape[TIAB] OR apes[TIAB] OR pan[TIAB] OR paniscus[TIAB] OR "pan paniscus"[TIAB] OR bonobo[TIAB] OR bonobos[TIAB] OR troglodytes[TIAB] OR "pan troglodytes"[TIAB] OR gibbon[TIAB] OR gibbons[TIAB] OR siamang[TIAB] OR siamangs[TIAB] OR nomascus[TIAB] OR symphalangus[TIAB] OR chimpanzee[TIAB] OR chimpanzees[TIAB] OR prosimians[TIAB] OR "bush baby"[TIAB] OR prosimian[TIAB] OR bush babies[TIAB] OR galagos[TIAB] OR galago[TIAB] OR pongidae[TIAB] OR gorilla[TIAB] OR gorillas[TIAB] OR pongo[TIAB] OR pygmaeus[TIAB] OR "pongo pygmaeus"[TIAB] OR orangutans[TIAB] OR pygmaeus[TIAB] OR lemur[TIAB] OR lemurs[TIAB] OR lemuridae[TIAB] OR horse[TIAB] OR horses[TIAB] OR pongo[TIAB] OR equus[TIAB] OR cow[TIAB] OR calf[TIAB] OR bull[TIAB] OR chicken[TIAB] OR chickens[TIAB] OR gallus[TIAB] OR quail[TIAB] OR bird[TIAB] OR birds[TIAB] OR quails[TIAB] OR poultry[TIAB] OR poultries[TIAB] OR fowl[TIAB] OR fowls[TIAB] OR reptile[TIAB] OR reptilia[TIAB] OR reptiles[TIAB] OR snakes[TIAB] OR snake[TIAB] OR lizard[TIAB] OR lizards[TIAB] OR alligator[TIAB] OR alligators[TIAB] OR crocodile[TIAB] OR crocodiles[TIAB] OR turtle[TIAB] OR turtles[TIAB] OR amphibian[TIAB] OR amphibians[TIAB] OR amphibia[TIAB] OR frog[TIAB] OR frogs[TIAB] OR bombina[TIAB] OR salientia[TIAB] OR toad[TIAB] OR toads[TIAB] OR "epidalea calamita"[TIAB] OR salamander[TIAB] OR salamanders[TIAB] OR eel[TIAB] OR eels[TIAB] OR fish[TIAB] OR fishes[TIAB] OR pisces[TIAB] OR catfish[TIAB] OR catfishes[TIAB] OR siluriformes[TIAB] OR arius[TIAB] OR heteropneustes[TIAB] OR sheatfish[TIAB] OR perch[TIAB] OR perches[TIAB] OR percidae[TIAB] OR perca[TIAB] OR trout[TIAB] OR trouts[TIAB] OR char[TIAB] OR chars[TIAB] OR salvelinus[TIAB] OR "fathead minnow"[TIAB] OR minnow[TIAB] OR cyprinidae[TIAB] OR carps[TIAB] OR carp[TIAB] OR zebrafish[TIAB] OR zebrafishes[TIAB] OR goldfish[TIAB] OR goldfishes[TIAB] OR guppy[TIAB] OR guppies[TIAB] OR chub[TIAB] OR chubs[TIAB] OR tinca[TIAB] OR barbels[TIAB] OR barbus[TIAB] OR pimephales[TIAB] OR promelas[TIAB] OR "poecilia reticulata"[TIAB] OR mullet[TIAB] OR mullets[TIAB] OR seahorse[TIAB] OR seahorses[TIAB] OR mugil curema[TIAB] OR atlantic cod[TIAB] OR shark[TIAB] OR sharks[TIAB] OR catshark[TIAB] OR anguilla[TIAB] OR salmonid[TIAB] OR salmonids[TIAB] OR whitefish[TIAB] OR whitefishes[TIAB] OR salmon[TIAB] OR salmons[TIAB] OR sole[TIAB] OR solea[TIAB] OR "sea lamprey"[TIAB] OR	6306321<br><br>OR<br><br>322905	15:29:42	27/08/2018

Data base	Descriptors	Items Found	Time	Date
	lamprey[TIAB] OR lampreys[TIAB] OR pumpkinseed[TIAB] OR sunfish[TIAB] OR sunfishes[TIAB] OR tilapia[TIAB] OR tilapias[TIAB] OR turbot[TIAB] OR turbots[TIAB] OR flatfish[TIAB] OR flatfishes[TIAB] OR sciuridae[TIAB] OR squirrel[TIAB] OR squirrels[TIAB] OR chipmunk[TIAB] OR chipmunks[TIAB] OR suslik[TIAB] OR susliks[TIAB] OR vole[TIAB] OR voles[TIAB] OR lemming[TIAB] OR lemmings[TIAB] OR muskrat[TIAB] OR muskrats[TIAB] OR lemmus[TIAB] OR otter[TIAB] OR otters[TIAB] OR marten[TIAB] OR martens[TIAB] OR martes[TIAB] OR weasel[TIAB] OR badger[TIAB] OR badgers[TIAB] OR ermine[TIAB] OR mink[TIAB] OR minks[TIAB] OR sable[TIAB] OR sables[TIAB] OR gulo[TIAB] OR gulos[TIAB] OR wolverine[TIAB] OR wolverines[TIAB] OR minks[TIAB] OR mustela[TIAB] OR llama[TIAB] OR llamas[TIAB] OR alpaca[TIAB] OR alpacas[TIAB] OR camelid[TIAB] OR camelids[TIAB] OR guanaco[TIAB] OR guanacos[TIAB] OR chiroptera[TIAB] OR chiropteras[TIAB] OR bat[TIAB] OR bats[TIAB] OR fox[TIAB] OR foxes[TIAB] OR iguana[TIAB] OR iguanas[TIAB] OR xenopus laevis[TIAB] OR parakeet[TIAB] OR parakeets[TIAB] OR parrot[TIAB] OR parrots[TIAB] OR donkey[TIAB] OR donkeys[TIAB] OR mule[TIAB] OR mules[TIAB] OR zebra[TIAB] OR zebras[TIAB] OR shrew[TIAB] OR shrews[TIAB] OR bison[TIAB] OR bisons[TIAB] OR buffalo[TIAB] OR buffaloes[TIAB] OR deer[TIAB] OR deers[TIAB] OR bear[TIAB] OR bears[TIAB] OR panda[TIAB] OR pandas[TIAB] OR "wild hog"[TIAB] OR "wild boar"[TIAB] OR fitchew[TIAB] OR fitch[TIAB] OR beaver[TIAB] OR beavers[TIAB] OR jerboa[TIAB] OR jerboas[TIAB] OR capybara[TIAB] OR capybaras[TIAB]) NOT medline[subset])			
	#3 Plants<br><br>(“Plants, Medicinal”[MeSH Terms] OR “Plant Extracts”[MeSH Terms] OR “Phytotherapy”[MeSH Terms] OR “Herbal Therapy”[TIAB] OR “Herb Therapy”[TIAB] OR “Drugs, Chinese Herbal”[MeSH Terms] OR “Chinese Plant Extracts”[TIAB] OR “Herbal Drug”[TIAB] OR “Herbal extract”[TIAB] OR “Plant Preparations”[MeSH Terms] OR “Herbal Preparation”[TIAB] OR “Plant Bark”[MeSH Terms] OR “plant leaves”[MeSH Terms] OR “plant leaf”[TIAB] OR “seeds extract”[TIAB] OR “bark extract”[TIAB] OR “leaves extract”[TIAB] OR “root extract”[TIAB] OR “fruits extract”[TIAB] OR “flowers Extract”[TIAB] OR “Plant Oils”[MeSH Terms] OR “Vegetable Oils”[TIAB])	294910	15:30:47	27/08/2018
	#4 Wound<br><br>(“Wound Healing”[MeSH terms] OR “Regeneration”[MeSH terms])	206236	15:31:52	27/08/2018
	#5 Bone<br><br>("bone remodeling"[MeSH Terms] OR "bone and bones"[MeSH Terms] OR "bone density"[MeSH Terms] OR "Skeleton"[MeSH Terms] OR "bone remodeling"[TIAB] OR "bone and bones"[TIAB] OR "bone density"[TIAB] OR "Skeleton"[TIAB] OR "Bone turnover"[TIAB] OR "Bone Mineral content"[TIAB] OR "Bone Loss"[TIAB])	826743	15:32:34	27/08/2018
	**Total: #1OR#2 and #3 and #4 and #5**	**174**	**15:34:58**	**27/08/2018**
**Data base**	**Descriptors**	**Items Found**	**Time**	**Date**
**Scopus**	#1Plants<br><br>(TITLE-ABS-KEY(“Plants, Medicinal”) OR TITLE-ABS-KEY(“Plant Extracts”) OR TITLE-ABS-KEY(“Phytotherapy”) OR TITLE-ABS-KEY(“Herbal Therapy”) OR TITLE-ABS-KEY(“Herb Therapy”) OR TITLE-ABS-KEY(“Drugs, Chinese Herbal”) OR TITLE-ABS-KEY(“Chinese Plant Extracts”) OR TITLE-ABS-KEY(“Herbal Drug”) OR TITLE-ABS-KEY(“Herbal extract”) OR TITLE-ABS-KEY(“Plant Preparations”) OR TITLE-ABS-KEY(“Herbal Preparation”) OR TITLE-ABS-KEY(“Plant Bark”) OR TITLE-ABS-KEY(“plant leaves”) OR TITLE-ABS-KEY(“plant leaf”) OR TITLE-ABS-KEY(“seeds extract”) OR TITLE-ABS-KEY(“bark extract”) OR TITLE-ABS-KEY(“leaves extract”) OR TITLE-ABS-KEY(“root extract”) OR TITLE-ABS-KEY(“fruits extract”) OR TITLE-ABS-KEY(“flowers Extract”) OR TITLE-ABS-KEY(“Plant Oils”) OR TITLE-ABS-KEY(“Vegetable Oils”))	409,019	15:37:30	27/08/2018
	#2 Wound<br><br>(TITLE-ABS-KEY(“Wound Healing”) OR TITLE-ABS-KEY(Regeneration) OR TITLE-ABS-KEY(repair))	838,555	15:38:14	27/08/2018
	#3 Bone<br><br>(TITLE-ABS-KEY(“bone remodeling”) OR TITLE-ABS-KEY(“bone”) OR TITLE-ABS-KEY(“bone density”) OR TITLE-ABS-KEY(“Skeleton”) OR TITLE-ABS-KEY("Bone turnover") OR TITLE-ABS-KEY("Bone Mineral content") OR TITLE-ABS-KEY("Bone Loss"))	1,383,568	15:39:21	27/08/2018
	#4 Animal Experiment<br><br>Scopus gives us the option to limit the Experimental Animal research on keywords	108	15:42:46	27/08/2018

	#5 Language<br>Scopus gives us the option to limit the Experimental Animal research on keywords	100	15:45:13	27/08/2018
	**Total: #1 and #2 and #3 and #4 and #5**	**100**	**15:46:00**	**27/08/2018**

Standardized descriptors were defined by MeSH algorithm and non-MeSH descriptors were characterized by the [TIAB] algorithm, which was also used to recover recently published but non indexed studies (*in process*). A previously published and optimized animal filter was applied in PubMed search interface. The same search filters used for disease and intervention were adapted for Scopus. The own Scopus animal filter (Keyword – animals [limit to]) was used in this database.
